# The Safety of Cold Versus Hot Snare Polypectomy in Polyps 10-20 mm: A Systematic Review and Meta-Analysis

**DOI:** 10.7759/cureus.58462

**Published:** 2024-04-17

**Authors:** Saeed Ali, Neelam Khetpal, Munazza Fatima, Sana Hussain, Asad Ali, Muhammad Ali Khan, Christopher Childs, Muhammad K Hasan

**Affiliations:** 1 Division of Gastroenterology and Hepatology, University of Illinois Chicago, Chicago, USA; 2 Department of Hospital Medicine, Hartford Hospital, Hartford, USA; 3 Department of Anesthesiology, State University of New York (SUNY) Upstate Medical University Hospital, Syracuse, USA; 4 Department of Medicine, Khyber Teaching Hospital, Peshawar, PAK; 5 Department of Gastroenterology and Hepatology, State University of New York (SUNY) Upstate Medical University Hospital, Syracuse, USA; 6 Division of Gastroenterology and Hepatology, University of Texas MD Anderson Cancer Center, Houston, USA; 7 Hardin Library for the Health Sciences, University of Iowa, Iowa City, USA; 8 Department of Gastroenterology, Center for Interventional Endoscopy, AdventHealth Orlando, Orlando, USA

**Keywords:** pooled analysis, post-polypectomy bleeding, colorectal cancer, colon polyps, snare polypectomy

## Abstract

Colonoscopy remains the primary method for preventing colorectal cancer. Traditionally, hot snare polypectomy (HSP) was the method of choice for removing polyps larger than 5 mm. Yet, for polyps smaller than 10 mm, cold snare polypectomy (CSP) has become the favored approach. Lately, the use of CSP has expanded to include the removal of sessile polyps that are between 10 and 20 mm in size. Our systematic review and meta-analysis aimed to evaluate the safety of cold snare polypectomy (CSP) compared to hot snare polypectomy (HSP) for resecting polyps measuring 10-20 mm. We searched the Medical Literature Analysis and Retrieval System Online (MEDLINE), Embase, and Cochrane databases up to April 2020 to find studies that directly compared CSP to HSP for polyps larger than 10 mm. Our main focus was on assessing the risk of delayed bleeding after polypectomy; a secondary focus was the incidence of any adverse events that required medical intervention post procedure. Our search yielded three comparative studies, two observational studies, and one randomized controlled trial (RCT), together encompassing 1,193 polypectomy procedures. Of these, 485 were performed using CSP and 708 with HSP. The pooled odds ratio (OR) for post-polypectomy bleeding (PPB) was 0.36 (95% confidence interval {CI}: 0.02, 7.13), with a Cochran Q test P-value of 0.11 and an I^2^ of 53%. For the risk of any adverse events necessitating medical care, the pooled OR was 0.15 (95% CI: 0.01, 2.29), with a Cochran Q test P-value of 0.21 and an I^2^ of 35%. The quality of the two observational studies was deemed moderate, and the RCT was only available in abstract form, preventing quality assessment. Our analysis suggests that there is no significant difference in the incidence of delayed post-polypectomy bleeding or other adverse events requiring medical attention between CSP and HSP for polyps measuring 10-20 mm.

## Introduction and background

Colonoscopy remains the gold standard screening test for colon cancer prevention. Screening with colonoscopy has been shown to reduce the incidence [1-4] and mortality [1,3-5] of colorectal cancer by performing polypectomies of high-risk adenomas, thereby interrupting the adenoma-carcinoma sequence [1-3]. Hot snare polypectomy (HSP) has traditionally been the preferred technique for removing polyps, utilizing electrosurgical current through a snare to cut the polyp tissue. This method aims to reduce bleeding during the procedure by cauterizing the resected tissue. While HSP effectively stops bleeding immediately, it carries the risk of post-polypectomy bleeding (PPB), perforation, and post-polypectomy syndrome (PPS). Post-polypectomy bleeding rates vary from 0.04% to 7.8% [6-10], with an increased bleeding risk associated with larger polyps, polyps located on the right side of the colon, polyps with advanced histological features, and the use of antithrombotic medications [9,11-13]. Studies have found that 60% of HSP procedures cut into the deeper submucosa layer and 20% reach the muscularis propria layer, areas with larger and more numerous blood vessels [14]. Consequently, the risk of injuring arteries in the submucosal layer can be as high as 39%, contributing to post-polypectomy bleeding [15]. Additionally, the occurrence of perforation during HSP is reported to be between 1.4% and 1.5% [16,17].

More recently, polyp resection without electrocautery, called cold snare polypectomy (CSP), has gained favor. This preference is attributed to its efficiency in reducing the duration of the procedure and its perceived lower risk of complications, particularly delayed post-polypectomy bleeding (PPB). It has become the preferred method of polypectomy, particularly for small polyps [18]. Studies suggest that CSP has comparable efficacy and safety to HSP, particularly for polyps <10 mm in size [19-25]. A multicenter randomized controlled trial (RCT) reported more cases of delayed PPB in the HSP group compared to the CSP group [26]. Studies have also evaluated the safety of CSP versus HSP in polyps measuring >10 mm up to 20 mm. However, there is no consensus among endoscopists regarding the use of one modality over the other in polyps measuring >10 mm. Hence, we performed a systematic review and meta-analysis to assess and compare the safety profiles of these two techniques for removing polyps measuring 10-20 mm.

This article was presented as a meeting abstract at the American College of Gastroenterology Virtual Annual Scientific Meeting and Postgraduate Course on October 27, 2020.

## Review

Materials and methods

Data Sources and Search Strategy

This systematic review adhered to the Preferred Reporting Items for Systematic Reviews and Meta-Analyses (PRISMA) guidelines [27]. A skilled medical librarian (CAC) carried out the search strategy and literature search. Search strategies were initially formulated for Ovid Medical Literature Analysis and Retrieval System Online (MEDLINE) and then adjusted to align with the subject headings and keywords relevant to the Ovid Embase and the Cochrane databases, covering the period from their inception up to April 24, 2020. The following search terms were included: endoscopic mucosal resection, CSP versus HSP, cold snare polypectomy versus hot snare polypectomy, colon cancer screening, conventional polypectomy, polypectomy, post-polypectomy bleeding, tubular adenomas, colon polyps, colonoscopy, sigmoidoscopy, and colon perforation. The strategy employed wildcards to capture plural forms and spelling variations. There were no language limitations. Selection for full-text review was based on the relevance of titles and abstracts. Additional articles were identified through manual searches of references and related articles (backward snowballing). All identified records were imported into EndNote (Thomson Reuters, Toronto, Canada) for organization, and duplicate records were removed.

Inclusion and Exclusion Criteria

Two of the study's authors (SA and MAK) set the inclusion criteria, focusing on studies that compared HSP to CSP in polyps larger than 10 mm and had a minimum follow-up duration of 14 days. These studies could be observational studies or randomized controlled trials (RCTs), including patients over the age of 18 years. Studies had to report delayed PPB or adverse events after colonoscopy requiring medical attention. Studies were excluded if only polypectomies for <10 mm polyps were reported, and outcomes of interest (e.g., PPB and adverse events) were not reported. We also excluded studies reporting experimental data on animals, studies with no comparative data, case reports/series, and review articles. The titles and abstracts were independently assessed by two reviewers (NK and AA), who made their inclusion decisions based on the predefined eligibility criteria. In cases of disagreement, a third reviewer (MKH) was consulted, and a consensus was reached through discussion.

Data Extraction, Quality Assessment, and Statistical Analysis

Data from included studies were independently extracted by two reviewers (NK and SA) on prespecified datasheets. Extracted data included study design, country, year of publication, patient demographics, and adverse events including PPB. Following the full extraction of data, the extracted information was reviewed, and any differences in interpretation between the reviewers were resolved through discussion with a third reviewer (MAK), aiming for a unanimous decision.

The quality of the included studies was independently evaluated by two researchers (SA and MAK) using the Newcastle-Ottawa Scale (NOS) designed for observational studies. Should there be any differences in the assessment of study quality, these were resolved by involving a third researcher (MKH) to reach a consensus through discussion.

Our primary outcome of interest was the difference in delayed PPB and adverse events requiring medical intervention. The pooled odds ratio (OR) and the 95% confidence interval (CI) were determined and analyzed employing the DerSimonian random-effects model [28]. When zero outcome events were encountered in both arms, a continuity correction of 0.5 was applied. To evaluate heterogeneity among the studies, we employed the I^2^ statistics and the Cochran Q test. A Cochran Q test with a P-value of less than 0.1 and an I^2^ statistic exceeding 50% were indicative of significant heterogeneity. Although we initially intended to investigate publication bias, this analysis was not conducted because our review included fewer than 10 studies. The Comprehensive Meta-Analysis Software, version 3.0, from Biostat in Englewood, New Jersey, the USA, was used for conducting all the statistical analyses.

Results

**Figure 1:**
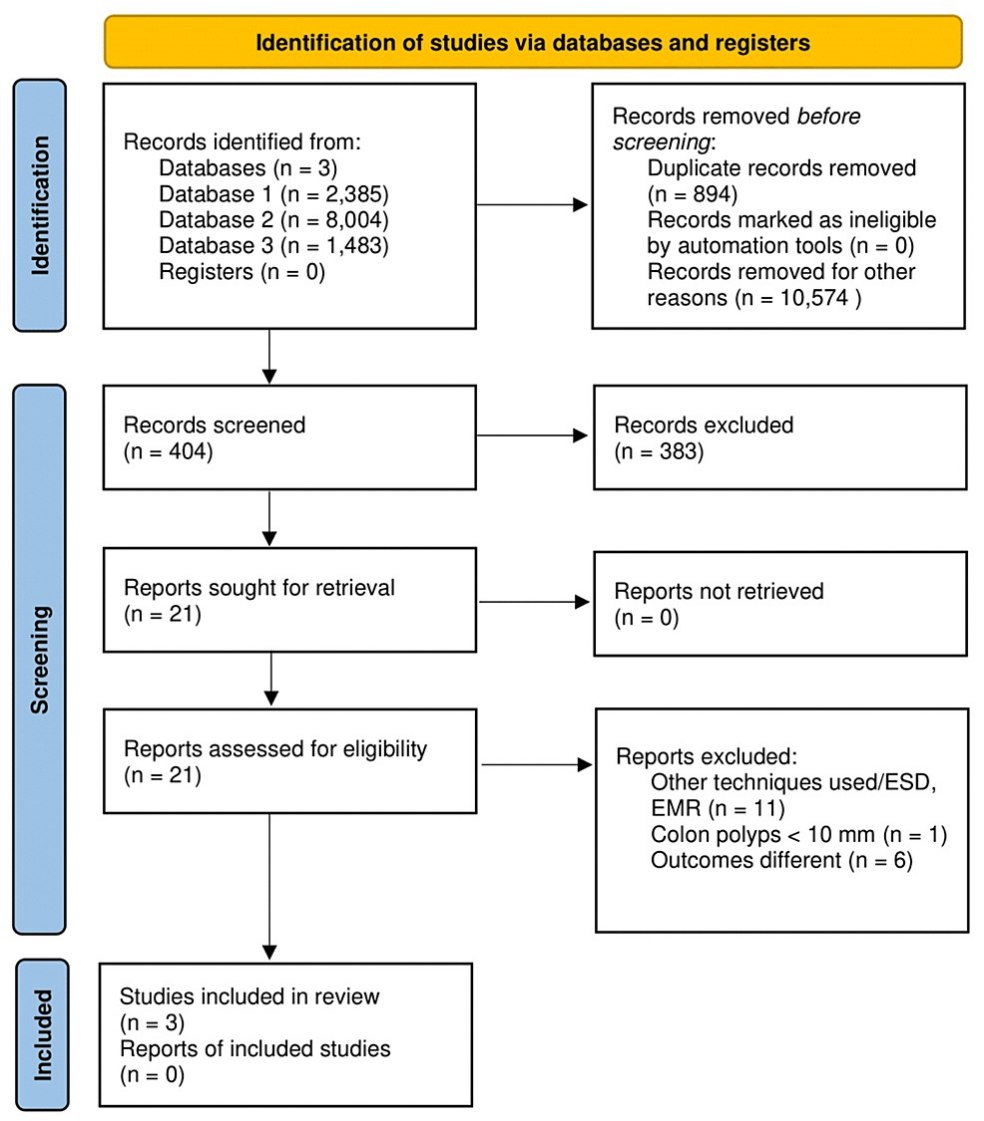
PRISMA flowchart PRISMA flow diagram showing the study selection process PRISMA, Preferred Reporting Items for Systematic Reviews and Meta-Analyses; ESD, endoscopic submucosal dissection; EMR, endoscopic mucosal resection

The initial search yielded 11,872 citations, with 10,978 left after removing duplicates. Following a review of titles and abstracts, 10,574 citations were excluded. No additional studies were found through bibliographic searches of the remaining articles. Consequently, out of 404 studies assessed, 401 were excluded for not meeting our inclusion criteria, leaving three studies (two observational studies and one randomized controlled trial) for inclusion in our systematic review and meta-analysis. The process of selecting these studies is depicted in Figure 1.

One study included polyps ranging from 5 to 20 mm, but only data for polyps measuring >10 mm were included in the analysis [29]. The remaining two studies exclusively included polyps 10-20 mm [30,31]. Using the NOS assessment, the study by Ket et al. [30] was rated as high quality, while that by Gessl et al. [29] was rated as moderate quality. The included RCT was in abstract form, and there was not enough methodology provided to gauge quality using the Cochrane tool for assessing the risk of bias.

A total of 1,193 polypectomies were included in this analysis: 485 CSP and 708 HSP. The total number of patients specified by two studies (Ket et al. [30] and Pillai et al. [31]) was 532. Two studies (Ket et al. [30] and Gessl et al. [29]) reported the histology of polyps as follows: a total of 352 sessile serrated adenomas (SSA; 156 HSP and 196 CSP) and 763 tubular adenomas (540 HSP and 223 CSP). Only one study reported complete resection rates [29]. Overall, tubular adenomas with low-grade dysplasia had complete resection rates of 84% and 81% with CSP and HSP, respectively. Likewise, tubular adenomas with high-grade dysplasia had resection rates of 51% and 70% with CSP and HSP, respectively. There was no statistical difference reported based on chi-square testing for complete resection rates.

**Figure 2:**
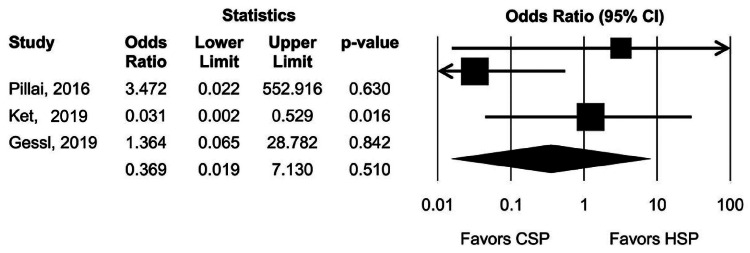
Pooled delayed post-polypectomy bleeding rate Gessl et al., 2019 [29]; Ket et al., 2019 [30]; Pillai et al., 2016 [31] CSP, cold snare polypectomy; HSP, hot snare polypectomy; CI, confidence interval

All studies reported the incidence of delayed PPB. The rates of delayed PPB were 0.2% and 1% with CSP and HSP, respectively. Pooled OR with 95% CI for delayed PPB was 0.36 (0.02, 7.13), with a Cochran Q test P-value of 0.11 and I^2^ of 53%. Pillai et al. also reported that 5% of CSP patients experienced immediate bleeding, which required the placement of endoscopic clips, while no patients undergoing HSP had immediate PPB (Figure 2) [31].

**Figure 3:**
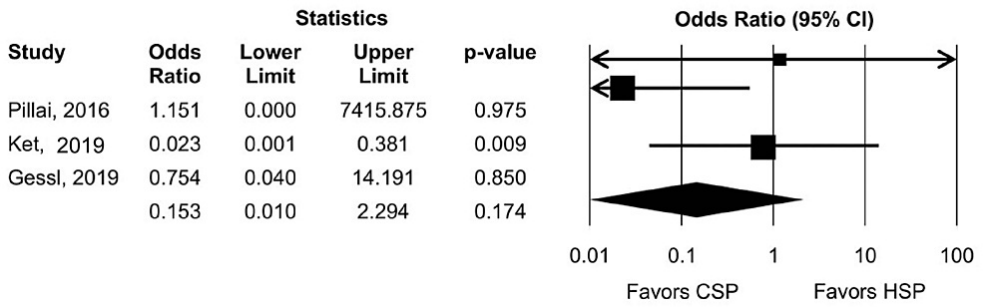
Pooled adverse event rate during follow-up Gessl et al., 2019 [29]; Ket et al., 2019 [30]; Pillai et al., 2016 [31] CSP, cold snare polypectomy; HSP, hot snare polypectomy; CI, confidence interval

The overall rates of post-polypectomy adverse events (excluding delayed bleeding) requiring hospitalization or additional procedures were 0% and 2% with CSP and HSP, respectively. Pooled OR with 95% CI was calculated to be 0.15 (0.01, 2.29). The Cochran Q test resulted in a P-value of 0.21, and the heterogeneity measure I^2^ was found to be 35% (Figure 3).

Discussion

Despite the growing body of evidence in favor of CSP for the management of colon polyps, practice among gastroenterologists remains highly variable. Shinozaki et al. compared the efficacy and safety of CSP versus HSP in 3,195 small colorectal polyps and found that CSP not only was associated with significantly shorter procedure time but also showed a trend toward lower incidence of delayed PPB [32]. HSP can result in eschar formation due to the use of electrosurgical current with an increased risk of delayed PPB, PPS, and perforation. The clinical guidelines from the European Society of Gastrointestinal Endoscopy (ESGE) advocate for the use of cold snare polypectomy (CSP) as the method of choice for removing small, diminutive polyps less than 9 mm in size [18].

In this systematic review and meta-analysis of 1,193 polypectomies, we did not find any difference in the risk of delayed PPB or other adverse events requiring hospitalization with either CSP or HSP. The incidences of delayed PPB and other major adverse events with CSP were 0.2% and 0%, respectively. Our results are consistent with the findings of Piraka et al., who found no incidence of adverse events in 73 patients with colon polyps measuring >10 mm [21]. A recent pooled analysis of single-arm, non-comparative studies evaluating the efficacy and safety of CSP in 522 polyps measuring >10 mm reported an adverse event rate of 1.1%. The rate of delayed PPB was 0.5%, while the rate of intra-procedural bleeding requiring endoscopic clipping was 0.7%. In a subgroup analysis of polyps measuring >20 mm, the risk of immediate bleeding requiring endoscopic intervention was 1.3% with no incidence of delayed PPB [33].

To our knowledge, this is the first systematic review and meta-analysis of comparative studies evaluating the safety of CSP versus HSP. Our investigation involved a thorough search of the literature, incorporating all pertinent studies. However, the strength of our analysis may be diminished by the intrinsic limitations associated with meta-analyses and the characteristics of the included studies, given that to date, only three comparative studies have been published. We did not encounter significant heterogeneity in our estimates. We could not pool complete resection rates and time duration for procedures as they were not uniformly reported by individual studies. Likewise, the risk of bleeding based on the location of polyps could not be evaluated due to limited data. Further, the use of anticoagulation and antiplatelet agents may affect delayed PPB, but such data were not provided by every study. However, we believe that anticoagulation and antiplatelet agents were managed before endoscopy, following guidelines of the American Society for Gastrointestinal Endoscopy and the European Society of Gastrointestinal Endoscopy, and would have been uniform for both CSP and HSP.

## Conclusions

In summary, our study demonstrates that CSP is as safe as HSP for the management of colon polyps ranging from 10 mm to 20 mm. The scope of our study was somewhat restricted in terms of analyzing delayed post-polypectomy bleeding in relation to specific polyp traits and assessing efficacy through complete resection rates, owing to the scarcity of available data. There is a need for randomized controlled trials (RCTs) to compare the effectiveness and safety of these techniques, which could potentially strengthen the case for preferring CSP.
